# Efficacy and tolerability of a 4-month ofloxacin-containing regimen compared to a 6-month regimen in the treatment of patients with superficial lymph node tuberculosis: a randomized trial

**DOI:** 10.1186/s12879-024-09511-w

**Published:** 2024-07-25

**Authors:** Syed Hissar, Banurekha Velayutham, Manoharan Tamizhselvan, Sridhar Rathinam, Chinnadurai Arunbabu, Jayanthi Bharathi Vidhya, Gurusamy Vargunapandian, Anandakrishnan Sundararajaperumal, Gomathi Narayan Sivaramakrishnan, Silambu Chelvi, Paranchi Murugesan Ramesh, Damodharan Arun, Sirasanambati Devarajulu Reddy, Paramasivam Paul Kumaran, Marimuthu Makesh Kumar, Dharuman Kalaiselvi, Luke Elizabeth Hanna, Hemanth Kumar, Alagarsamy Gowrisankar, Ramasamy Rajavelu, Lavanya Jayabal, Chinnayan Ponnuraja, Dhanaraj Baskaran

**Affiliations:** 1 Indian Council of Medical Research - National Institute for Research in Tuberculosis, No: 1, Mayor Sathyamoorthy road, Chetpet, Chennai, 600031 India; 2 Government Stanley Medical College Hospital, Chennai, India; 3 Rajiv Gandhi Government General Hospital, Chennai, India; 4 Kilpauk Medical College Hospital, Chennai, India; 5 Government Vellore Medical College Hospital, Vellore, India; 6 National Tuberculosis Elimination Programme, Chennai, India

**Keywords:** Tuberculosis, Extra pulmonary tuberculosis, Lymphadenitis, Biopsy, Ofloxacin

## Abstract

**Background:**

Tuberculosis (TB) lymphadenitis is the most common form of extra-pulmonary TB, and the treatment duration is six months. This non-inferiority based randomized clinical trial in South India evaluated the efficacy and safety of a four-month ofloxacin containing regimen in tuberculosis lymphadenitis (TBL) patients.

**Methods:**

New, adult, HIV-negative, microbiologically and or histopathologically confirmed superficial lymph node TB patients were randomized to either four-month oflaxacin containing test regimen [ofloxacin (O), isoniazid (H), rifampicin (R), pyrazinamide (Z) -2RHZO daily/ 2RHO thrice-weekly] or a six-month thrice-weekly control regimen (2HRZ, ethambutol/4RH). The treatment was directly observed. Clinical progress was monitored monthly during and up to 12 months post-treatment, and thereafter every three months up to 24 months. The primary outcome was determined by response at the end of treatment and TB recurrence during the 24 months post-treatment.

**Results:**

Of the 302 patients randomized, 298 (98.7%) were eligible for modified intention-to-treat (ITT) analysis and 294 (97%) for per-protocol (PP) analysis. The TB recurrence-free favourable response in the PP analysis was 94.0% (95% CI: 90.1–97.8) and 94.5% (95% CI: 90.8–98.2) in the test and control regimen respectively, while in the ITT analysis, it was 92.7% and 93.2%. The TB recurrence-free favourable response in the test regimen was non-inferior to the control regimen 0.5% (95% CI: -4.8-5.9) in the PP analysis based on the 6% non-inferiority margin. Treatment was modified for drug toxicity in two patients in the test regimen, while one patient had a paradoxical reaction.

**Conclusion:**

The 4-month ofloxacin containing regimen was found to be non-inferior and as safe as the 6-month thrice-weekly control regimen.

## Background

Tuberculosis (TB) lymphadenitis is the most common presentation of extra-pulmonary TB (EPTB), accounting for 30–40% of EPTB [1]. Earlier, under the Revised National Tuberculosis Control Programme (RNTCP) of India, patients with TB lymphadenitis (TBL) were treated with a thrice-weekly regimen (Category-I) with four drugs (Rifampicin (R), Isoniazid (H), Ethambutol (E), and Pyrazinamide (Z)) for the first two months, followed by two drugs (Rifampicin and Isoniazid) for the next four months [2]. The delivery of TB chemotherapy in the field would be much easier if the duration of therapy could be shortened without sacrificing efficacy. This requires drugs with potent bactericidal and/or sterilizing activity against *Mycobacterium tuberculosis (M.tb)*, which shortens the treatment duration.

The fluoroquinolone group of drugs has been demonstrated to have significant therapeutic potential in the management of TB [3]. These bactericidal drugs, which inhibit DNA gyrase, are highly active against *M.tb*, including strains resistant to first-line drugs [4, 5]. Ofloxacin has moderately high early bactericidal activity (EBA) compared to other anti-TB drugs [6]. Previous clinical trials in patients with sputum positive pulmonary TB reported that regimens of four- to five-month duration, in which Ofloxacin was substituted for Ethambutol for the first three months, could achieve high cure rates at the end of treatment and low relapse rates during follow-up [7]. Based on this finding and with the rationale that similar results could be achieved with paucibacillary conditions like lymph node TB, we evaluated the efficacy of a four-month ofloxacin containing regimen in terms of response at the end of treatment and TB recurrence up to 24 months post-treatment compared to the six months thrice-weekly regimen in newly diagnosed superficial lymph node TB patients. In addition, adverse drug reactions and paradoxical reactions were compared.

## Methods

We conducted an open-label, parallel arm, randomized controlled clinical trial from 2012 to 2022 (CTRI/2013/03/003481; Date:31/01/2013). Study was approved by the Scientific Advisory Committee and the Institutional Ethics Committee.

### Study participants

We enrolled newly diagnosed adult patients with histopathological or microbiological evidence of TB in the biopsy sample of the lymph node swelling or Fine Needle Aspiration Cytology (FNAC) of lymph node swelling from sites in Chennai, Madurai, and Vellore in South India based on the eligibility criteria. We excluded those weighing < 30 kg, evidence of pulmonary TB, mediastinal adenopathy in chest x-ray, other forms of extra-pulmonary TB, hepatic or renal disease, HIV infection, epilepsy, and pregnant or lactating women. Written informed consent was obtained from the study participants.

### Treatment regimens

Eligible participants were allocated in a ratio of 1:1 to either the test or the control regimen.

Test regimen: Rifampicin (R), Isoniazid (H), Pyrazinamide (Z), and Ofloxacin (O) daily for two months, followed by Rifampicin, Isoniazid, and Ofloxacin thrice-weekly for two months (2RHZO daily / 2RHO thrice-weekly) - Duration: four months.

Control regimen: Rifampicin (R), Isoniazid (H), Ethambutol (E), and Pyrazinamide (Z) thrice-weekly for two months, followed by Rifampicin and Isoniazid thrice-weekly for four months (2RHZE thrice-weekly / 4RH thrice-weekly) – Duration: six months.

Stratification was done on the basis of previous TB treatment (up to one month) and diabetes; either nil or any. Restricted random allocation sequences were generated using random number tables, and sequentially numbered sealed envelopes were used to assign the regimens.

The drug dosages were as follows: Ofloxacin – 600 mg (800 mg for body weight > 45 kg); Isoniazid – 300 mg (daily) or 600 mg (thrice-weekly); Rifampicin – 450 mg (600 mg for body weight > 60 kg); Pyrazinamide – 1500 mg; and Ethambutol – 1200 mg. During daily drug administration of the test regimen in the intensive phase, directly observed treatment was given for five days a week and the two weekend doses were self-administered. All the doses during the thrice-weekly treatment were supervised. Missed doses were compensated for up to 15 days of duration.

### Investigations

At baseline, the site, size, and number of lymph nodes, abscess, sinus formation, and biopsy site were documented. A general and systemic examination was done. Chest x-ray were done on all participants. For those with a productive cough and abnormal chest x-rays, a minimum of two sputum specimens were collected (1 overnight and 1 spot). The presence of epithelioid cells, caseous necrosis, and granulomatous lesions consistent with tuberculosis was considered histopathological evidence. Smears were graded using fluorescence microscopy, and cultures were graded by growth in Lowenstein Jensen (LJ) medium based on standard procedures [8]. A cartridge based nucleic acid amplification test (CBNAAT) was done in the FNAC/biopsy of a lymph node as per manufacturer procedure [9]. Lymph node biopsy specimens were subjected to mycobacterial culture using multiple culture media and *M.tb* identification by standard methods [10–12]. Drug susceptibility tests (DST) based on WHO recommendations were done by MIC method for H, R, E, and O and defined as in previous studies [13–17]. Patients diagnosed with multidrug resistant TB (MDR-TB) based on the culture results after enrolment, were withdrawn from the study and referred to TB programme for appropriate management. A complete hemogram, hepatic and renal function tests, random blood sugar, ELISA for HIV, urine routine, and gravindex (for women) were done.

Study participants were reviewed every month for clinical progress, examination of residual lymph nodes, systemic examination, and drug adverse effects. A chest x-ray was done at the end of two months and at the end of treatment. A sputum examination was done for those with a productive cough and an abnormal chest x-ray. A complete hemogram, hepatic and renal function tests, random blood sugar, urine routine, and gravindex (for women) were done every month up to four months. Study participants with favourable responses to treatment were followed up every month up to 12 months post-treatment and thereafter every 3 months up to 24 months for the evaluation of TB recurrence. Those with unfavourable responses to treatment or those with TB recurrences were treated as per the recommendations of the National TB Elimination Program (NTEP).

### Study outcomes

The response to treatment was determined by an independent assessor who was blinded to the treatment received by the study participants. The independent assessor evaluated the participants prior to the start of treatment, at the end of treatment, and during follow-up in cases of enlarged lymph nodes.

### Primary outcome

#### Status at the end of treatment

Favourable: regression of existing lymph nodes to less than 10 mm in diameter and absence of an appearance of new nodes, healing of existing sinus; and absence of the development of fresh sinuses.

Unfavourable: Any one of the following.

- There was no change in the size and number of lymph nodes and/or sinuses at the end of treatment.
- Appearance of new TB lymph nodes (exceeding 10 mm in diameter) at a site not affected initially at the end of three months of treatment or later.
- Development of TB at other sites at any time after the start of treatment.
- Positive sputum cultures (at least two) at any time after the start of treatment.

TB recurrence during follow-up in those with favorable responses at the end of treatment was based on any of the following:

- Appearance of new nodes or enlargement of existing nodes in which the diagnosis of TB is confirmed either by histopathology or bacteriology of the lymph node specimen.
- Development of TB at other sites at any time during follow-up.

### Secondary outcomes

Paradoxical reactions: Enlargement of existing nodes or appearance of new nodes or of sinuses and abscesses during or after anti-tuberculosis treatment in the absence of evidence of active disease or relapse or the presence of another diagnosis.

Adverse reactions to anti-TB drugs: Participants who developed adverse reactions attributable to drugs in the treatment regimens.

### Sample size and statistical analysis

Using the non-inferiority design with a margin of 6%, we assumed the efficacy of the control regimen to be 95%, with an alpha of 5%, and a power of 80%, we calculated 165 patients in each arm, a total of 330 patients in the trial. During the course of the trial, after treatment outcomes were available for 259 patients, and based on the observation that it was similar between the regimens, the sample size was recalculated as 290 to achieve the power of 80% to detect the difference.

Data was analysed using the SPSS 25.0 software (IBM Corp., Armon, NY, USA). The proportion of patients with favourable, unfavourable responses to treatment, TB recurrence, adverse drug reactions, and paradoxical reactions was compared between the test and control regimens. The analysis was done by both intention-to-treat and per-protocol analysis. Non-inferiority was defined if the upper limit of a one-sided 95% confidence interval (CI) of the percentage difference between the test and control regimen is less than 6% in the per-protocol analysis. Proportions were compared using the Chi-square test. A *P*-value ≤ 0.05 was considered significant.

## Results

### Patient population

**Fig. 1:**
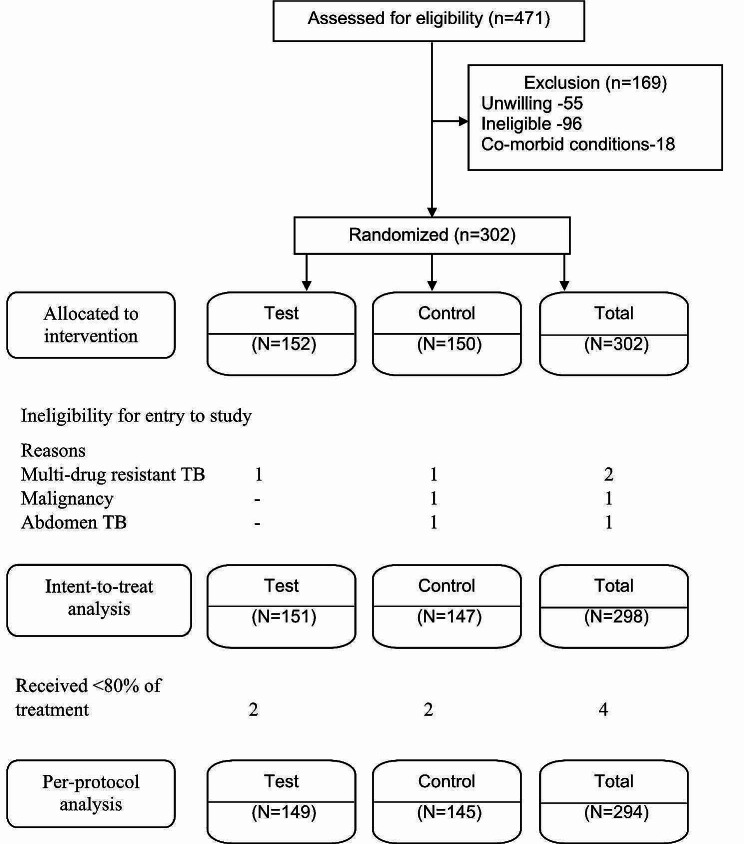
Flow diagram of patients from eligibility to analysis stages

Of the 471 patients screened, 302 were enrolled (Fig. 1). There were 298 patients, who were considered for the intention-to-treat analysis after excluding 4 (MDR-TB-2; Malignancy-1; Abdominal TB -1) patients. Of the 298, excluding four who received < 80% of treatment, 294 were considered for the per-protocol analysis (Fig. 1). Females constituted 206 (69.1%) of the 298 patients in the intention-to treat analysis, the mean age was 30.3 (SD = 9.2) years, and the mean BMI was 23.1 (SD = 5.0) kg/m^2^. There were 142 (47.7%) who were aged ≥ 30 years, 143 (48.0%) had BMI > 23 kg/m^2^, 296 (99.3%) had no previous anti-TB treatment, and six (2.0%) were diabetics (Table 1). The baseline characteristics of age, sex, BMI, previous anti-TB treatment, and diabetes were similar in both regimens. There were 288 (96.6%) patients with cervical lymph node involvement. Overall, 49 (17.6%) were smear positive from lymph node biopsy specimens, and 113 (86.3%) had *M.tb* detected by CBNAAT in lymph node FNAC samples. Lymph node culture positivity for *M.tb* was 122 of 146 (83.6%) in the test regimen and 98 of 142 (69.0%) in the control regimen (*p* = 0.009). Overall, there were 195 (65.4%) patients with lymph node *M.tb* cultures susceptible to H, R, E, and O.

**Table 1:** Baseline characteristics of 298 TB lymphadenitis patients enrolled in the study- modified intention-to-treat group

Patient characteristics		Test	Control	Overall	*P* value
		*N* = 151	*N* = 147	*N* = 298	
		*n* (%)	*n* (%)	*n* (%)	
Sex	Female	103 (70.1)	103 (68.2)	206 (69.1)	0.729
	Male	44 (29.9)	48 (31.8)	92 (30.9)	
Age (years)	< 30	74 (50.3)	82 (54.3)	156 (52.3)	0.493
	≥ 30	73 (49.7)	69 (45.7)	142 (47.7)	
Body mass Index (Kg/m^2^)	< 16	9 (6.1)	5 (3.3)	14 (4.7)	0.550
	16–18.49	16 (10.9)	22 (14.6)	38 (12.8)	
	18.5–22.9	52 (35.4)	51 (33.8)	103 (34.6)	
	>=23	70 (47.6)	73 (48.3)	143 (48.0)	
Previous anti-TB	Nil	146 (99.3)	150 (99.3)	296 (99.3)	1.000
Treatment	< 15 days	1 (0.7)	1 (0.7)	2 (0.7)	
Diabetes	Yes	2 (1.4)	4 (2.6)	6 (2.0)	0.684
	No	145 (98.6)	147 (97.4)	292 (98.0)	
Lymph node involvement	Cervical	146 (96.7)	142 (96.6)	288 (96.6)	0.911
	Axillary	2 (1.3)	3 (2.0)	5 (1.7)	
	Cervical and Axillary	2 (1.3)	1 (0.7)	3 (1.0)	
	Supraclavicular	1 (0.7)	1 (0.7)	2 (0.7)	

		*N* = 142	*N* = 137	*N* = 279	
Lymph node smear for	Positive	26 (18.3)	23 (16.8)	49 (17.6)	0.739
*M.tb* from biopsy / FNAC *(N = 279)*	Negative	116 (81.7)	114 (83.2)	230 (82.4)	

		*N* = 66	*N* = 65	*N* = 131	
Lymph node CBNAAT for	*M.tb* detected	59 (89.4)	54 (83.1)	113 (86.3)	0.296
*M.tb* from biopsy / FNAC *(N = 131)*	*M.tb* not detected	7 (10.6)	11 (16.9)	18 (13.7)	

		*N* = 146	*N* = 142	*N* = 288	
Lymph node	Positive	122 (83.6)	98 (69.0)	220 (76.4)	0.009
culture for *M.tb* from biopsy / FNAC	Negative	20 (13.7)	40 (28.2)	60 (20.8)	
(*N* = 288)	*NTM*	4 (2.7)	4 (2.8)	8 (2.8)	

		*N* = 151	*N* = 147	*N* = 298	
Lymph node smear and/or CBNAAT and/or culture for *M.tb* from biopsy / FNAC *(N = 279)*	Positive	134 (88.7)	112 (76.2)	246 (82.6)	0.004
	Negative	17 (11.3)	35 (23.8)	52 (17.4)	

		*N* = 122	*N* = 98	*N* = 220	
Drug susceptibility	Susceptible to H, R, E, O	104 (68.9)	91 (61.9)	195 (65.4)	0.078
profile of *M.tb* cultures	Any resistance				
*(N = 220)*	Resistant to H	6 (4.0)	3 (2.0)	9 (3.0)	
	Resistant to O	11 (7.3)	4 (2.7)	15 (5.0)	
	Resistant to EO	1 (0.7)	-	1 (0.3)	

### Status at the end of treatment

There were 143 of 151 (94.7%) in the test regimen and 140 of 147 (95.2%) in the control regimen with favourable response at the end of treatment in the intention-to-treat analysis (Table 2). In the per-protocol analysis of 294 patients, 143 of 149 (96.0%) in the test regimen and 140 of 145 (96.6%) in the control regimen had favourable responses (Table 2). Treatment was changed for hepatic and gastrointestinal toxicity in two patients in the test regimen. There was one patient in the control regimen who committed suicide during the 2nd month of treatment (non-TB death).

**Table 2:** Status at the end of treatment and TB recurrence during follow-up in per-protocol and modified intention-to-treat populations

End of treatment status	Per-protocol analysis *N* = 294		Modified intention-to-treat analysis *N* = 298	
	Test	Control	Test	Control
	*N* = 149	*N* = 145	*N* = 151	*N* = 147
Favourable response to treatment n (%) (95% CI)	143 (96.0%) (92.8 to 99.1)	140 (96.6%) (93.6 to 99.5)	143 (94.7%) (91.1 to 98.3)	140 (95.2%) (91.8 to 98.7)
Unfavourable response	4 (2.7%)	4 (2.8%)	4 (2.6%)	4 (2.7%)
No change in lymph node size	3 (2.0%)	4 (2.8%)	3 (2.0%)	4 (2.7%)
Appearance of new nodes	1 (0.7%)	0	1 (0.7%)	0
Death due to other Non-TB cause	0	1 (0.7%)	0	1 (0.7%)
Treatment changed for drug toxicity	2 (1.3%)	0	2 (1.3%)	0
Withdrawn from treatment (Missed > 1 month of treatment continuously)	-	-	2 (1.3%)	2 (1.4%)
TB recurrence in those with favourable response to treatment- Enlargement /appearance of new nodes	3 (2.0%)	3 (2.1%)	3 (2.0%)	3 (2.0%)
TB recurrence free favourable response among the total patients n (%) (95% CI)	140 (94.0%) (90.1 to 97.8)	137 (94.5%) (90.8 to 98.2)	140 (92.7%) (88.6 to 96.9)	137 (93.2%) (89.1 to 97.3)
% Difference between the test and control regimen (95% CI)	0.5% (95% CI: -4.8 to 5.9)		0.5% (95% CI: -5.3 to 6.3)	

### TB recurrence

There were 6 patients (3 each in the test and control regimen) who had TB recurrence with the appearance of new nodes. Of the 6 patients with TB recurrence, *M.tb* was detected in Xpert MTB/RIF in 4 patients (2 each in the test and control regimen), and rifampicin resistance was not detected. The remaining 2 patients had histopathology of FNAC suggestive of TB. Patients with TB recurrences were unwilling to undergo a biopsy of the lymph node specimen. TB recurrence occurred in the second month in one patient and in the fourth month in two patients post-treatment in the test regimen. The TB recurrence for 3 patients was at 3rd, 4th, and 14th months post-treatment in the control regimen.

TB recurrence-free favourable response in the per-protocol analysis was 94.0% (95% CI: 90.1–97.8) and 94.5% (95% CI: 90.8–98.2) in the test and control regimen respectively, while in the intention-to-treat analysis it was 92.7% (95% CI: 88.6–96.9) and 93.2% (95% CI: 89.1–97.3) (Table 2). The difference in the TB recurrence free proportion of success was 0.5% (95% CI: -4.8–5.9%), thus the test regimen was non-inferior to the control regimen.

### Adverse drug reactions

The adverse drug reactions due to anti-TB drugs in 298 patients in the intention-to-treat analysis are shown in Table 3. There were 19 (12.6%) patients with adverse drug reactions (ADR) in the test regimen, compared to 6 (4.1%) in the control regimen (OR: 3.38; 95% CI: 1.3–8.7; *p* = 0.012). Musculoskeletal (Arthralgia-8, Myalgia-1) was observed in nine patients in the test regimen. Gastro-intestinal symptoms (nausea, vomiting, and epigastric pain) were observed in five and three patients in the test and control regimen respectively. The ADRs were mild in both regimens except in 2 patients in the test regimen for whom treatment had to be modified for hepatic and gastrointestinal toxicity.

**Table 3:** Adverse reactions attributable to anti-TB drugs in 298 patients (Modified intention-to-treat group)

	Test	Control	Total	OR (95%CI)
	*N* = 151	*N* = 147	*N* = 298	*p*-value
Patients with any adverse drug reaction n (%) (95% CI)	19 (12.6%) (7.3 to 17.9)	6 (4.1%) (0.9 to 7.3)	25 (8.4%)	3.38 (1.3 to 8.7) 0.012
**Adverse drug reaction**	*N* = 23	*N* = 8	*N* = 31	
Arthralgia/Myalgia	9	-	9	
Gastrointestinal	5	3	8	
Giddiness	3	1	4	
Peripheral neuropathy	-	2	2	
Cutaneous	5	1	6	
Hepatic	1	-	1	
Flu-like syndrome	-	1	1	

OR: Odds ratio; CI: Confidence interval

### Paradoxical reaction

There was one patient in the test regimen who had an enlargement of the lymph node during the second month of treatment. FNAC was done, and culture grew Staphylococcus Aureus. The patient was treated with antibiotics and there was resolution of the lymph node.

## Discussion

Our study has shown that in the treatment of superficial lymph node TB, the four-month part daily ofloxacin containing regimen (2RHZO daily / 2RHO thrice-weekly) is non-inferior to the six-month intermittent regimen (2RHEZ thrice-weekly / 4RH thrice-weekly). Clinical trials of superficial lymph node TB in adults are limited. An earlier study in children and adults comparing six-month regimen with Isoniazid and Rifampicin with the six-month twice-weekly regimen of Isoniazid and Rifampicin with Pyrazinamide in the intensive phase documented 94% and 96% favourable responses at the end of 36 months [18]. The National TB Elimination Programme of India (NTEP) currently recommends six-month daily regimen for the treatment of TB lymphadenitis [19]. The comparator regimen in our study is not the currently recommended daily six months regimen but the six-month thrice-weekly regimen which was the standard of care up to 2016. Nevertheless, the four -months ofloxacin containing regimen with the daily intensive phase has given a TB recurrence-free favourable response of 94% in the per-protocol analysis and is non-inferior to the control six-month thrice-weekly regimen. This indicates that superficial lymph node TB can be treated with a shorter four -months quinolone containing regimen.

Resistance to fluoroquinolone is a matter of concern considering the use of the drug in first-line treatment. The first national anti-tuberculosis drug resistance survey in India conducted during 2014–2016 documented 3.72% ofloxacin resistance in new pulmonary patients [20]. We observed an overall ofloxacin resistance of 5% in the present study in *M.tb* cultures of lymph node biopsy specimens, with nearly 8% resistance in the test regimen. Despite that, the patients had a favourable responses of > 90% with the ofloxacin containing four-month shorter regimen. However, in this era of drug susceptibility test (DST)-guided treatment, upfront testing for fluoroquinolone resistance with molecular testing is crucial prior to using quinolone-containing regimens in TB treatment. This is possible in pulmonary TB but is challenging in the context of extra-pulmonary TB specimens.

Overall, there were 8.4% of patients with any adverse drug reaction. There is a lack of data on adverse drug reactions among patients treated for TB lymphadenitis. The adverse drug reactions to first-line anti-TB drugs in various Indian studies among pulmonary TB patients ranged from 8.37 to 69% [21]. Adverse drug reactions due to anti-TB drugs were higher in the test regimen compared to the control regimen. However, treatment modification was required only in 2 (1.3%) of patients treated with the test regimen. Earlier studies in pulmonary TB with daily pyrazinamide in the intensive phase of TB treatment have reported arthralgia [7, 22]. We observed arthralgia in 8 patients in the test regimen with daily pyrazinamide compared to none in the control regimen, which is similar to the earlier studies. A paradoxical reaction was observed in one patient in our study. On the contrary, earlier studies have reported paradoxical reactions of about 20–35% in patients with TB lymphadenitis [23, 24]. The incidence of paradoxical reactions observed in our study was much lower than anticipated, which could be due to the fact that the majority of our study patients (82%) had an excision biopsy of the enlarged lymph node for the diagnosis of TB. Non-inclusion of people living with HIV (PLHIV) in this study could also be a possible reason. Nevertheless, though the study patients were monitored closely, there could be a possibility of non-reporting of symptoms by the patients.

Female preponderance was observed in our study population (69.1% females vs. 30.9% males). Previous studies from India, Africa and United States have documented a higher female-to-male ratio in lymph node TB (1.4:1) [23–25]. About 82.6% of our study population had a BMI of > 18.5 kg/m^2^. This is contrary to the observation in pulmonary TB wherein low BMI and undernutrition are predominant [26, 27]. The observation of female preponderance, lower magnitude of undernutrition in TB lymphadenitis requires further evaluation.

The limitations of the study include that it was not multi-centric and had a long recruitment period. In addition, the six-month thrice-weekly control regimen which is not the current standard of care for lymph node TB, was used as a comparator in the trial. When the study was initiated in 2012, the Revised National TB Control Programme (RNTCP) of India offered a Category-I regimen for new TB patients (2HRZE in the intensive phase and 4 h in the continuation phase, given thrice -weekly). We used the Category-I regimen as the control since it was the standard of care then.

To conclude, this study has shown that superficial lymph node TB can be successfully treated with a 4-month ofloxacin containing regimen (2RHZO daily / 2RHO thrice-weekly). The effectiveness of this regimen needs to be studied in field settings with appropriate treatment adherence mechanisms to ensure drug compliance. Shorter TB treatment regimens are beneficial to both patients and the health system.

## Data Availability

All data generated or analysed during this study are included in this published article.

## Abbreviations

TBL: tuberculosis lymphadenitis
O: ofloxacin
H: isoniazid
R: rifampicin
Z: pyrazinamide
E: ethambutol
ITT: intention-to-treat
PP: per-protocol
CI: confidence interval
EBA: early bactericidal activity
FNAC: fine needle aspiration cytology
DST: drug susceptibility tests
LJ: Lowenstein Jensen
CBNAAT: cartridge based nucleic acid amplification test
NTEP: National TB Elimination Program
ADR: adverse drug reaction
